# The effect of 10-min dispatch-assisted cardiopulmonary resuscitation training: a randomized simulation pilot study

**DOI:** 10.1186/s12245-020-00287-9

**Published:** 2020-06-11

**Authors:** Hidetada Fukushima, Hideki Asai, Tadahiko Seki, Keisuke Takano, Francesco Bolstad

**Affiliations:** 1 Department of Emergency and Critical Care Medicine, Nara Medical University, Shijo-cho 840, Kashihara City, Nara 634-8522 Japan; 2 Department of Emergency, Nara Prefecture General Medical Center, Shichijo-Nishimachi 2-897-5, Nara City, 630-8581 Japan; 3 Clinical English, Nara Medical University, Shijo-cho 840, Kashihara City, Nara 634-8522 Japan

**Keywords:** Cardiac arrest, Prehospital, Resuscitation training

## Abstract

**Background:**

Immediate bystander cardiopulmonary resuscitation (CPR) is essential for survival from sudden cardiac arrest (CA). Current CPR guidelines recommend that dispatchers assist lay rescuers performing CPR (dispatch-assisted CPR (DACPR)), which can double the frequency of bystander CPR. Laypersons, however, are not familiar with receiving CPR instructions from dispatchers. DACPR training can be beneficial for lay rescuers, but this has not yet been validated. The aim of this study was to determine the effectiveness of simple DACPR training for lay rescuers.

**Methods:**

We conducted a DACPR simulation pilot study. Participants who were non-health care professionals with no CPR training within 1 year prior to this study were recruited from Nara Medical University Hospital. The participants were randomly assigned to one of the two 90-min adult basic life support (BLS) training course groups: DACPR group (standard adult BLS training plus an additional 10-min DACPR training) or Standard group (standard adult BLS training only). In the DACPR group, participants practiced DACPR through role-playing of a dispatcher and an emergency caller. Six months after the training, all subjects were asked to perform a 2-min CPR simulation under instructions given by off-duty dispatchers.

**Results:**

Out of the 66 participants, 59 completed the simulation (30 from the DACPR group and 29 from the Standard group). The CPR quality was similar between the two groups. However, the median time interval between call receipt and the first dispatch-assisted compression was faster in the DACPR group (108 s vs 129 s, *p* = 0.042).

**Conclusions:**

This brief DACPR training in addition to standard CPR training can result in a modest improvement in the time to initiate CPR. Future studies are now required to examine the effect of DACPR training on survival of sudden CA.

## Background

Sudden cardiac arrest (CA) is a leading cause of death in industrialized nations. Bystander cardiopulmonary resuscitation (CPR) can increase the chance of survival from out-of-hospital CA (OHCA) [1–3]. Bystander CPR, however, is initiated only 10–40% in the USA, Europe, and Asia [2, 4, 5].

Dispatchers can help untrained emergency callers identify CA and instruct them to start prompt chest compression [6]. It is reported that dispatch-assisted CPR (DACPR) may double the frequency of CPR initiation by bystanders [7]. Familiarizing laypersons with DACPR may allow lay rescuers to perform CPR more quickly. However, the effectiveness of adding DACPR training to standard CPR training, in terms of strengthening bystander CPR, has not been deeply investigated.

In order to highlight the effect of DACRP training for lay rescuers, we conducted a randomized pilot study for DACPR training.

## Methods

### Study design

We conducted a parallel randomized pilot study to compare DACPR quality among participants from two CPR trainings: a standard CPR training and a standard CPR training with an additional DACPR training. We recruited non-health care professionals with no CPR training experience within 1 year prior to this study from Nara Medical University Hospital.

### CPR trainings

The training sessions were held at Nara Medical University. The first cohort was given standard adult basic life support (BLS) training (Standard group), while the second cohort was given standard adult BLS training with an additional 10-min DACPR training (DACPR Group). In the DACPR group, participants learned DACPR through caller-dispatcher role-playing with CPR manikins and a template for CPR instruction at the end of the training. Both training courses were 90 min. The participants were assigned to one of the adult BLS trainings randomly scheduled based on a computer program. The training sessions took place in a room at the university, where a maximum of 10 people can learn CPR. All the participants were blinded to their allocations. Six months after the trainings, all participants were invited to the DACPR simulation via phone.

### DACPR simulation

We conducted DACPR simulation 6 months after the training. In this simulation, the participants performed a single rescuer scenario in a small room at Nara Medical University. In this room, there was a manikin (Laerdal Resusci Anne manikin with Skill Reporting System) lying on a hard floor and a cordless extension phone on a small table. Neither an AED nor other rescuers were present for this simulation. After being given a list of simple instructions (Appendix 1), the participants entered the room as if they happened to find someone (CPR manikin) unconscious on the floor; they then performed CPR under the instruction of dispatchers. Nine dispatchers with at least 1 year of emergency dispatch experience took part in this simulation. All dispatchers were blinded to the participants’ allocations between the two cohorts. The study dispatchers provided CPR instructions along with the standard DACPR guidelines prepared by the Japanese Fire and Disaster Management Agency [8] after giving the simulation instruction (Appendix 2). The study participants and dispatchers communicated through a closed telephone line system for emergency call training. The study investigators recorded the time and ensured that 2 min was kept for each simulation. After performing the simulation, each participant was offered a $10 value gift card as an incentive for the simulation.

### Data collection and outcome

Data for chest compression performance (mean depth [mm], mean rate [cpm, compression per minute], hand position [%]) were collected through the Laerdal Skill Reporting System®. All simulations were recorded on video cameras (SONY HDR-AS 200V). The following time intervals were measured: [1] the call to identify the need for CPR, [2] the call to start CPR instruction, and [3] the call to start the first chest compression. The outcome of this study was the effect of DACPR on time interval between call receipt and the first chest compression, and the quality of chest compressions.

### Statistics

Since this study was a pilot study, the target sample size was 30 in each group with reference to the previous studies [9–14].

Continuous variables were described as median and interquartile range (IQR), and categorical variables were described as number (percentages). We used Mann-Whitney *U* test for continuous variables and chi-square test for categorical variables. Two-tailed *p* values less than 0.05 were considered as significant. Data analysis was done by SPSS ver. 22.0 (SPSS Inc., Chicago, IL, USA).

## Results

**Fig. 1:**
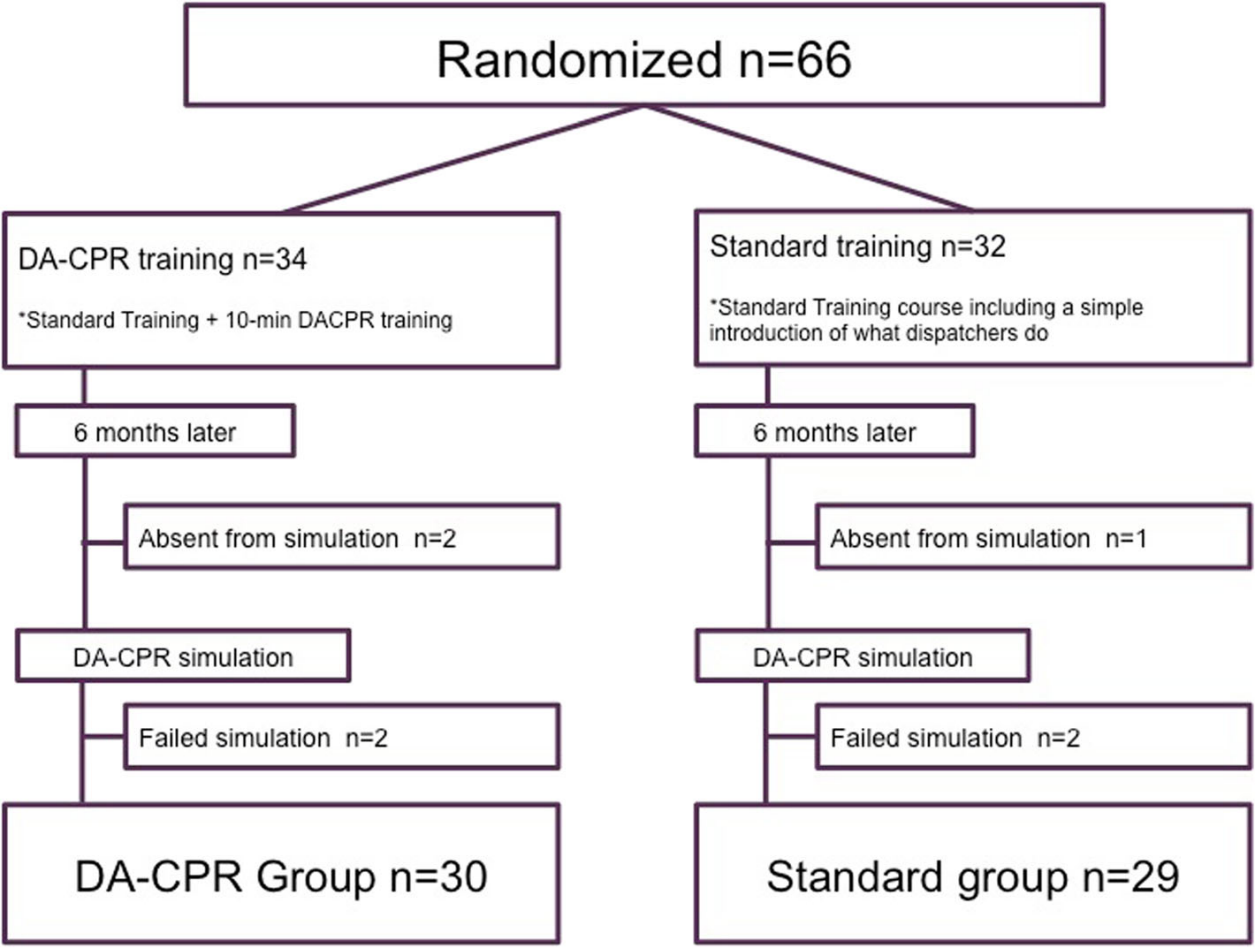
Study population

A total of 66 participants, aged 20s to 50s, were recruited and randomly assigned to the study groups (*N* = 34; 17 males, DACPR group and *N* = 32; 15 males, Standard group). After 6 months, 59 participants completed the DACPR simulation (*N* = 30; 15 males, DACPR group and *N* = 29; 13 males, Standard group, Fig. 1). The results are shown in Table 1. The overall chest compression performances were similar between the two groups. The average compression depth in both groups did not meet the recommended standard of 5 cm. The median time intervals between the call and the dispatchers’ recognition of CA, and call to the start of CPR instruction, were prompt in both groups, but relatively faster in the DACPR group (23.5 s vs 27 s, *p* = 0.187; 79.5 s vs 93 s, *p* = 0.069, respectively). The median time intervals between the call receipt and the start of chest compressions was significantly faster in the DACPR group (108 s) compared to the Standard group (129 s, *p* = 0.042) (Table 2).

**Table 1:** Chest compression qualities

	DACPR group	Standard group	*p* value
	(*N* = 30)	(*N* = 29)	
Correct hand position (> 90%), *n* (%)	23 (76.7)	21 (72.4)	0.708^a^
Compression depth, mm	41.5 (35.8–49.0)	45 (34.0–51.0)	0.504^b^
Compression rate, cpm	99 (77.8–108.3)	104 (91.0–106.5)	0.727^b^
Compression fraction, %	100 (97.9–100.0)	100 (97.9–100.0)	0.873^b^

Continuous values are expressed as median (interquartile)

*CA* cardiac arrest, *DACPR* dispatch-assisted cardiopulmonary resuscitation, *cpm* compressions per minute

^a^Chi-square test

^b^Mann-Whitney *U* test

**Table 2:** DACPR time intervals between groups

	DACPR group	Standard group	*p* value^a^
	(*N* = 30)	(*N* = 29)	
Call to CA recognition, s	23.5 (14.0–41.0)	27 (20.5–42.5)	0.187
Call to instruction, s	79.5 (62.8–104.8)	93 (83.0–102.0)	0.069
Call to DACPR, s	108 (89.8–136.5)	129 (106.5–148.0)	0.042

Continuous values are expressed as median (interquartile)

*CA* cardiac arrest, *DACPR* dispatch-assisted cardiopulmonary resuscitation

^a^Mann-Whitney *U* test

## Discussion

In this simulation study, participants from the DACPR training group demonstrated faster initiation of CPR with dispatcher instructions 6 months after training, when compared to the standard CPR training group. This result indicates that this brief DACPR training for lay rescuers has a potential benefit on CPR education.

The median time interval from call to first chest compression was 21 s faster in the DACPR group than in the Standard CPR group (108 s vs 129 s, *p* = 0.042). Kim et al. conducted a similar randomized simulation study to compare the effects of DACPR training to standard CPR training on the quality of DACPR. Consistently, Kim et al. also found that participants who underwent DACPR training started chest compressions 20 s faster than those who underwent standard CPR training, in a simulation that occurred 6 months after the initial training [9]. A possible explanation for this time reduction is that participants in the DACPR group were more familiar with the dispatcher instructions for CPR. For Standard CPR group participants, this DACPR simulation was their first experience to follow the instruction. Whether this 21-s reduction in the start of CPR represents a meaningful change in terms of the clinical outcomes of CA now warrants investigation. Studies have shown that it takes 3 to 4 min to start CPR when callers receive dispatch instruction in real-life situations [15, 16]. DACPR training for lay rescuers might be of clinical significance since the chance of survival with good neurological outcome for sudden CA victims decreases by 7–10% every minute without CPR [17].

Other key elements of chest compression such as median depth, rate, fraction, and the proportion of correct hand positions were similar in the two groups. Among these key elements, chest compression depth was suboptimal in both groups. Several simulation studies on layperson CPR training have shown that the quality of chest compressions rarely meet the recommended standard [10, 11], even with dispatch assistance [9, 12]. Dispatch CPR instruction to achieve optimal chest compression depths needs further studies.

This study has several limitations. First, the sample size is small and the results are difficult to generalize. Second, this simulation was conducted at just 6 months after training; as such, the long-term effects of DACPR training remain to be investigated. Future studies that look at skill retention after 12 months or the effect of repeated DACPR training are thus warranted. Finally, the findings of our study might not be generalized to the entire population as our study cohort predominantly included young participants in their 20s to 50s. Most CA patients and callers are likely to be elderly in residential settings [18] and might not be able to perform DACPR promptly.

## Conclusions

This pilot study suggests that DACPR training for 10 min in addition to standard CPR training can result in a modest improvement in the time to initiate CPR. Future studies are now required to examine the effect of DACPR training on survival of sudden CA.

## Data Availability

The datasets used in the current study are available from the corresponding author on reasonable request.

## Appendix 1

**Instruction for Subjects**

Please perform this simulation as if you were in a real emergency situation.

If you find a man (manikin) lying on the floor when you enter this room, act as you find a real person on the floor.

YOU ARE the ONLY PERSON in this room.

YOU CAN’T FIND ANYBODY.

Call the person on the floor.

If not responsive, DO WHAT YOU THINK YOU HAVE TO DO until We say ‘STOP’.

AED is not available in this room.

You may find a phone.

Emergency number is 119 in here.

If you feel uncomfortable or pain in the back, arms, elbows or hands, please notify us.

Now, you’ve heard ‘BANG’ behind this door.

ARE YOU READY?

HERE WE GO!

## Appendix 2

**Instruction for dispatchers**

When you receive a call from the study subjects, act as you usually do. Except for the following;

You do not have to ask the caller where the address is. (This simulation is conducted as a private residential setting through a landline.)

When you realized that the call was cardiac arrest,

1. Ask callers whether the patient is on a hard surface floor on the back.
2. Ask callers to activate the speaker phone function.
3. Ask callers to open the patient’s bear chest.
4. And then start instruction for chest compression as you usually do.
5. DO NOT INSTRUCT RESCUE BREATHING.
6. Keep instructing for high quality compression until the simulation ends.
7. This simulation ends two minutes after the caller started chest compression.

## Abbreviations

CA: Cardiac arrest
OHCA: Out-of-hospital cardiac arrest
CPR: Cardiopulmonary resuscitation
DACPR: Dispatch-assisted cardiopulmonary resuscitation
EMS: Emergency medical services
